# Evaluation of the Anticancer Effects of DODP on Gene Expression and Oxidative Stress in Gastric Cancer: An Integrated Docking, Bioinformatics, and Experimental Approach

**DOI:** 10.3390/life16040534

**Published:** 2026-03-24

**Authors:** Tugba Agbektas, Hakki Coskun, Husnu Cagri Genc, Gulcihan Cinar Kaya, Ayca Tas, Kenan Goren, Alakbar Huseynzada, Ruslan Guliyev, Ulviyya Hasanova, Savas Kaya, Alejandro Morales-Bayuelo, Yavuz Silig

**Affiliations:** 1 Department of Food Processing Technologies Services, Yıldızeli Vocational School, Sivas Cumhuriyet University, 58140 Sivas, Türkiye; tubaagbektas@cumhuriyet.edu.tr; 2 Department of General Surgery, Faculty of Medicine, Sivas Cumhuriyet University, 58140 Sivas, Türkiye; hacoskun@cumhuriyet.edu.tr (H.C.); cagrigenc@cumhuriyet.edu.tr (H.C.G.); 3 Department of Medical Services and Techniques, Medical Laboratory Techniques, Yıldızeli Vocational School, Sivas Cumhuriyet University, 58140 Sivas, Türkiye; glchncnr96@gmail.com; 4 Department of Biochemistry, Faculty of Medicine, Sivas Cumhuriyet University, 58140 Sivas, Türkiye; aycatas@cumhuriyet.edu.tr (A.T.); ysilig@cumhuriyet.edu.tr (Y.S.); 5 Department of Organic Chemistry, Faculty of Arts and Sciences, Kafkas University, 36100 Kars, Türkiye; kenangoren49@gmail.com; 6 Industrial Chemistry Research Laboratory (ICRL), Baku State University, Z. Khalilov 33, Baku AZ 1148, Azerbaijan; alakbar.huseynzada1117@gmail.com; 7 Geotechnological Problems of Oil, Gas and Chemistry Scientific Research Institute (GPOGC SRI), Azerbaijan State Oil and Industry University, Baku AZ 1148, Azerbaijan; ruslandjan01@gmail.com; 8 Islamic World Educational, Scientific and Cultural Organization (ICESCO) Biomedical Materials Department, Baku State University, Z. Khalilov 33, Baku AZ 1148, Azerbaijan; u.alimammad@gmail.com; 9 Department of Chemistry, Faculty of Science, Sivas Cumhuriyet University, 58140 Sivas, Türkiye; 10 Grupo Genoma, Escuela de Medicina, Universidad del Sinú, Seccional Cartagena, Cartagena 3660300, Colombia; amorales@unisinucartagena.edu.co

**Keywords:** heterocyclic compound, *CD274 (PD-L1)*, apoptosis, cell cycle, antioxidant defense, LDH

## Abstract

(1) Background: Gastric cancer (GC) remains a major global health challenge due to its high heterogeneity and aggressive progression. The discovery of novel bioactive molecules with anticancer properties has, therefore, become a critical research focus. In this study, we synthesized and characterized 4,4′-(5,8-dioxa-2,11-diazadodecane-1,11-diene-1,12-diyl)diphenol (DODP) and evaluated its anticancer potential using molecular docking, bioinformatics, and experimental analyses. (2) Methods: The chemical structure of DODP was confirmed through ^1^H and ^13^C NMR spectroscopy. Molecular docking was conducted to examine the interaction of DODP with apoptosis and cell cycle-related proteins (*TP53*, *MDM2*, and *MYC*) and the immune checkpoint marker *CD274 (PD-L1)*. Cytotoxicity against AGS GC cells was determined using the MTT assay at concentrations ranging from 0.01 to 50 µM, and gene expression alterations were analyzed by quantitative polymerase chain reaction (qPCR) and bioinformatics evaluation. (3) Results: NMR data verified the successful synthesis of DODP. The docking results indicated strong binding affinity, especially with *TP53* and *CD274*. DODP showed notable cytotoxicity after 72 h of exposure and induced upregulation of *TP53*, *MYC*, and *CD274* and downregulation of *MDM2* in AGS cells. Although the patterns were consistent with cell-based and bioinformatic analyses, significant discriminatory ability in blood samples was observed only for *MYC* (AUC = 0.651; *p* = 0.044). (4) Conclusions: DODP influenced apoptosis-associated transcriptional responses in GC, offering early mechanistic evidence that should be evaluated in more comprehensive biological models.

## 1. Introduction

GC remains one of the leading causes of cancer-related mortality worldwide; however, its incidence has markedly declined in recent years due to the implementation of effective primary and secondary prevention strategies [1]. Gastric carcinomas account for nearly 90% of all gastric malignancies, whereas the remainder includes lymphomas, gastrointestinal stromal tumors, leiomyosarcomas, and neuroendocrine tumors [2]. Clinically and epidemiologically, gastric carcinomas are categorized into cardia and noncardia types. GC is a multifactorial disease influenced by environmental, dietary, and genetic factors [3]. Established risks include Helicobacter pylori infection, low socioeconomic status, excessive intake of salted and smoked foods, low fruit and vegetable consumption, tobacco and alcohol use, obesity, low physical activity, and gastroesophageal reflux disease. Current findings suggest that obesity and low physical activity, particularly excessive consumption of processed and salty foods, further compound these risks [4]. Additionally, radiation exposure, positive family history, and hereditary predisposition further contribute to disease development [5,6]. Chronic mucosal inflammation may progress to atrophy and metaplasia, detectable through endoscopic evaluation. Diffuse-type GC is highly associated with impaired E-cadherin expression and, unlike intestinal-type tumors, is less frequently preceded by well-defined precancerous lesions, although it remains linked to atrophic gastritis [7]. Despite significant advancements in cancer therapeutics, chemotherapy-related adverse effects such as nephrotoxicity, neurotoxicity, and ototoxicity underscore the need for novel anticancer compounds with improved safety and specificity [8,9,10]. In this context, metal complexes derived from Schiff bases have attracted considerable interest due to their promising pharmacological and antimicrobial properties, as well as their applicability in drug design and analytical chemistry [11,12,13]. Particularly, recently synthesized azomethine-containing molecules have demonstrated potent anticancer activity with enhanced tumor selectivity [14]. The cell cycle is tightly regulated to maintain genomic stability, where cyclin-dependent kinases (CDKs) and cyclins act as positive regulators, and CDK inhibitors function as negative regulators, suppressing tumor progression [15]. Oncogenic mutations enable malignant transformation by promoting uncontrolled proliferation and evading programmed cell death [16]. Apoptosis, regulated primarily through caspase activation, plays a critical role in cellular homeostasis, and its dysregulation contributes to the pathogenesis of cancer and other life-threatening disorders [17]. Understanding these molecular pathways is, therefore, essential for the development of new therapeutic strategies. However, current GC treatment modalities, such as platinum-based chemotherapy, fluoropyrimidines, and immune checkpoint inhibitors, still suffer from significant limitations, including poor target specificity, heterogeneous treatment response, dose-limiting toxicity, and the rapid development of molecular resistance [18]. These gaps in this therapeutic approach highlight the need for versatile small molecules capable of simultaneously modulating multiple cancer-related metabolic pathways [19]. DODP is particularly important in this context because it was designed to activate the TP53–MDM2–MYC–PD-L1 axis, which is underrepresented in current treatment strategies [20]. Therefore, DODP may offer complementary advantages by restoring apoptotic signaling, reducing oncogenic activity, and affecting immune checkpoint regulation. In this context, DODP is thought to address significant shortcomings in current GC therapies [21].

The aim of this study was to synthesize a novel heterocyclic compound (DODP) and evaluate its potential anticancer effects against GC by elucidating its interactions with key proteins involved in apoptosis, cell cycle regulation, and immune response through molecular docking, bioinformatics, experimental analyses, and enzyme activity assay.

## 2. Materials and Methods

### 2.1. Synthesis of DODP

**Figure 1:**
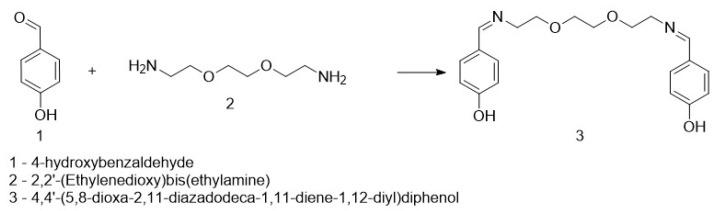
Synthesis of the targeted compound.

An amount of 0.1 g of 4-hydroxybenzaldehyde was dissolved in 5 mL of acetonitrile, followed by the addition of 0.1 mL of 2,2′-(ethane-1,2-diylbis(oxy))bis(ethan-1-amine) under stirring. After some time, a white suspension appeared. The reaction mixture was stirred for 2 h, after which the formed precipitate was filtered and rinsed several times with acetonitrile. The obtained product was identified as 4,4′-(5,8-dioxa-2,11-diazadodecane-1,11-diene-1,12-diyl)diphenol (Figure 1). The molecular weight of DODP (C_20_H_24_N_2_O_4_) is 356.41 g·mol^−1^, and all concentrations used in biological assays were standardized to µM based on this value.

### 2.2. Molecular Docking and ADMET Analysis

In this study, the potential binding affinities of the synthesized 4,4′-(5,8-dioxa-2,11-diazadodecane-1,11-diene-1,12-diyl) diphenol (DODP) compound with the biological target proteins associated with GC, TP53, MDM2, MYC, and PD-L1 (CD274) were investigated by molecular modeling approaches. All calculations were performed using the Schrödinger Maestro Molecular Modeling platform (version 11.8) [22]. The three-dimensional structure of the compound was optimized using the LigPrep module; in this process, different conformations were generated by considering possible ionization states, tautomeric structures, and stereoisomers. The crystal structures of the target proteins were obtained from the Protein Data Bank (PDB) database, and using the Protein Preparation Wizard tool, hydrogen atoms of the structures were added, missing side chains were completed, and water molecules outside 5 Å were removed [23]. Active sites were determined by reference to co-crystallized ligands and binding pockets defined in the literature; Glide Grid areas were created around these regions. Molecular docking was performed in the Glide module using the SlipDocking algorithm, which also takes protein flexibility into account. The docking results were evaluated based on binding energies and types of intermolecular interactions (hydrogen bonds, π–π stacking, hydrophobic interactions, etc.). The most suitable conformations were ranked according to their GlideScore values. Interaction analyses and visualizations of ligand–protein complexes were performed in both 2D and 3D formats using the Discovery Studio Visualizer (version 2016) [24]. In addition, the pharmacokinetic and toxicological properties of DODP were evaluated using the ADMETlab 2.0 online platform, and drug-like properties of the compound were predicted based on absorption, distribution, metabolism, elimination, and toxicity (ADMET) parameters.

### 2.3. Chemical Reactivity Context

Research in this field has revealed a well-established correlation between quantum similarity and descriptors related to chemical reactivity [25,26]. Both quantum similarity and density functional theory (DFT) leverage the density function as a fundamental component in the analysis of similarity indices. Specifically, the Coulomb index can be linked to electronic factors that impact chemical reactivity. Conceptual density functional theory (CDFT) focuses on the global and local reactivity of the atomic and molecular chemical systems in light of simplified approaches and electronic structural principles. Chemical potential measures the tendency of electrons to deviate from the equilibrium system, and chemical hardness evaluates a chemical species’ resistance to altering its electronic configuration. For the mathematical definition of the global reactivity indices, such as chemical potential (µ), electronegativity (χ), hardness (η), and softness (σ), CDFT presents the following formulae [27,28,29,30].

(1) $$\mu =-\chi ={\left[\frac{\partial E}{\partial N}\right]}_{\nu (r)}=-\left[\frac{I+A}{2}\right]$$

(2) $$\eta ={\left[\frac{\partial \mu }{\partial N}\right]}_{\nu (r)}={\left[\frac{\partial^{2}E}{\partial N^{2}}\right]}_{\nu (r)}=I-A$$

(3) σ=1/η

Here, E, N, I, and A represent the total electronic energy, total number of electrons, ionization energy, and electron affinity of the studied chemical systems, respectively.

The mathematical definition of the electrophilicity index (ω) is related to the stabilization energy of a system when it becomes saturated by electrons from the external environment [31].

(4) $$\omega =\chi^{2}/2\eta =\mu^{2}/2\eta$$

The ionization energy and electron affinity of the studied compound were predicted with the help of Koopmans’ theorem (KT), giving frontier orbital energy (HOMO and LUMO orbital energies)-based equations as [32]

(5) $$I=-E_{HOMO}$$

(6) $$A=-E_{LUMO}$$

In this research, the local reactivity descriptors under consideration were the Fukui functions. Equations (5) and (6) illustrate the system’s electronic density response to variations in the global charge, representing the derivative of the electronic density concerning the electron count under a consistent external field [33].

(7) $$f^{+}\left(\vec{r}\right)\approx {\left|LUMO\left(\vec{r}\right)\right|}^{2}$$

(8) $$f^{-}\left(\vec{r}\right)\approx {\left|HOMO\left(\vec{r}\right)\right|}^{2}$$

The terms and are utilized to represent nucleophilic and electrophilic attacks, respectively. This strategy incorporates both global and local reactivity descriptors to assess quantum similarity within the molecular set. All computations were executed using the B3LYP method with the 6-311XXG(d,p) basis set, an improvement over the 6-311G(d,p) basis set [34]. This enhancement enables calculations of electronegativity, hardness, reactivity indices, and frontier molecular orbitals at a quality level comparable to larger basis sets such as Aug-cc-pVQZ and Aug-cc-pV5Z. The Gaussian 16 package was employed in conjunction with this method/basis set combination [35].

### 2.4. Bioinformatics Analysis

The expression levels of the *CD274*, *TP53*, *MDM2*, and *MYC* genes were comprehensively analyzed using GTEx (https://gtexportal.org/home/), Kaplan–Meier Plotter (https://kmplot.com/analysis/ (accessed on 11 November 2025)), Gene Networks (HumanBase) (https://hb.flatironinstitute.org/gene/7442 (accessed on 11 November 2025)), GEPIA 2 (http://gepia2.cancer-pku.cn/#degenes (accessed on 11 November 2025)), UALCAN (https://ualcan.path.uab.edu/analysis.html (accessed on 11 November 2025)), TIMER 2.0 (http://timer.cistrome.org/), Enrichr-KG (https://maayanlab.cloud/enrichr-kg (accessed on 11 November 2025)), The Human Protein Atlas (https://www.proteinatlas.org/), and STRING databases (https://string-db.org/).

### 2.5. Sample Collection and Participants

Peripheral blood samples used in this study were collected from the Department of General Surgery, the Faculty of Medicine, Sivas Cumhuriyet University Training and Research Hospital (Sivas, Türkiye). A total of 60 participants were enrolled, comprising 30 patients diagnosed with GC and 30 age- and gender-matched healthy controls. All participants completed a structured questionnaire that included information on demographic characteristics, smoking habits, alcohol consumption, and family history of cancer. Written informed consent was obtained from each participant prior to inclusion in this study. Individuals with a prior history of cancer, ongoing medication use, or any chronic illness were excluded. The control group consisted of healthy volunteers without a history of malignancy or chronic disease. The clinical diagnosis of GC was histopathologically confirmed by an experienced pathologist. All blood samples were collected and processed in the laboratory where the experimental procedures were conducted.

### 2.6. Ethical Approval

The study protocol was reviewed and approved by the Sivas Cumhuriyet University Non-Interventional Clinical Research Ethics Committee (approval no.: 2023-05/03). Written informed consent was obtained from all participants prior to sample collection, and signed consent forms were securely archived. Ethical approval was granted for the human study, whereas the cell culture experiments did not require separate ethical approval.

### 2.7. Statistical Analysis

Statistical analyses were performed using the SPSS software (Version 22.0, IBM Corp., Armonk, NY, USA) and the GraphPad Prism software (Version 9.0, GraphPad Software, San Diego, CA, USA). The data were first tested for normality using the Shapiro–Wilk test. For comparisons between two groups with normally distributed data, Student’s *t*-test was applied to determine the significance of differences in mean qPCR values. One-way analysis of variance (ANOVA) followed by Tukey’s post hoc test was used for multiple comparisons among normally distributed groups. *p* < 0.05 was considered statistically significant. For data not conforming to a normal distribution, non-parametric tests such as the Mann–Whitney U test, Kruskal–Wallis test, and Chi-square (χ^2^) test were used to evaluate differences between groups. All graphical representations, including expression profiles and comparative plots, were generated using GraphPad Prism for enhanced visualization and clarity.

### 2.8. Cell Culture and In Vitro Cytotoxicity (MTT Assay)

AGS cells were cultured in Dulbecco’s modified Eagle’s medium (DMEM) supplemented with fetal bovine serum (FBS) and penicillin and maintained at 37 °C in a CO_2_ incubator. Once the cells reached 70–80% confluence, they were passaged into 75 cm^2^ flasks. Cells adhering to the flasks were detached using 2 mL of trypsin/EDTA solution and seeded into 96-well plates at a density of 1 × 10^5^ cells/well. The synthesized compound was applied at concentrations ranging from 0.01 to 50 µM, and the cells were incubated for 24, 48, and 72 h. The MTT assay was performed with three biological replicates (n = 3). DMSO (vehicle control) and a blank control were included as reference conditions. After incubation, 10 µL of MTT solution was added to each well and further incubated, followed by removal of the MTT reagent and addition of 100 µL of DMSO per well. The absorbance was measured at 570 nm, and IC_50_ values were calculated using GraphPad Prism 7.

### 2.9. Cell Morphology

AGS cells were seeded into plates at a density of 5 × 10^5^ cells/well, and 1 μM of the compound was added to each well. Morphological changes were examined using an imaging device (ZEISS Axio Vert.A1) at 20× magnification.

### 2.10. RNA Isolation from Cell Culture and Blood Samples

AGS cells were first grown in 25 cm^2^ vials and passaged in six-well plates once they reached a sufficient cell density. Compound application was performed according to the IC_50_ dose determined by the GraphPad Prism 7 software, and after the incubation period, RNA was isolated from the cells using the standard protocol of the RNeasy Plus Mini Kit. Following the same methodological approach, venous blood samples from the volunteers were collected in 4 mL EDTA tubes and converted to RNA according to the kit procedure. The purity and concentration of the isolated RNA were assessed using the 260/280 absorbance ratio on the NanoDrop device, and the quality range of the samples was confirmed to be 1.8–2.0.

### 2.11. cDNA Synthesis

cDNA was synthesized from the isolated RNA according to the manufacturer’s instructions provided with the synthesis kit.

### 2.12. qPCR Analysis

The expression levels of *TP53*, *MDM2*, *MYC*, and *PD-L1* were quantified by qPCR using the RT^2^ SYBR Green qPCR Master Mix. Gene expression was evaluated using the ΔΔ_CT_ method through the Qiagen Data Analysis Center (https://dataanalysis2.qiagen.com/pcr (accessed on 11 November 2025)) and further analyzed with Rotor-Gene 6000 and the RT^2^ Profiler qPCR Array Data Analysis software (version 3.5).

### 2.13. Validation of Reference Gene and qPCR Conditions

All qPCR reactions were performed using three independent biological replicates, and each sample was run in technical duplicates. *GAPDH* was used as the housekeeping gene based on its established stability in AGS and GC studies. In our dataset, *GAPDH* Ct values showed minimal variation across experimental groups (SD < 0.5–1 Ct), supporting its suitability for normalization. The specificity of amplification was confirmed by melt-curve analysis, which demonstrated a single sharp peak for each target without evidence of non-specific amplification or primer–dimer formation. The primer efficiency values for all gene targets (*TP53*, *MDM2*, *MYC*, *CD274*, and *GAPDH*) were within the acceptable range of 90–110%. The mean Ct values from technical duplicates were used for gene expression quantification. The relative mRNA levels were calculated using the 2^−∆∆CT^ method after normalization to *GAPDH*. The fold change value (fold change = 2^−∆∆CT^) indicates high gene expression when greater than one and low gene expression when less than one. When the fold change value (2^−∆∆CT^) is greater than one, it equals the fold regulation value; when less than one, the fold regulation value is the negative inverse of the fold change value.

### 2.14. Determination of Antioxidant Levels

#### 2.14.1. Determination of Glutathione (GSH)

GC cells were seeded into six-well plates and incubated. Following incubation, the compound was applied to the cells, which were then incubated for an additional 48 h. After treatment, the cells were detached using trypsin/EDTA solution, and 250 µL of the culture medium was collected. Glutathione levels were determined according to the protocol described by Giustarini et al. [36].

#### 2.14.2. Determination of Glutathione-S-Transferase (GST)

GST is an enzyme responsible for detoxifying numerous compounds generated by reactive oxygen species (ROS)-induced damage. After incubation of AGS cells with the compound, 250 µL of the culture medium was collected, and GST activity was assessed using the protocol of Ghelfi et al. [37].

### 2.15. Catalase (CAT) Determination

CAT activity is associated with oxidative stress and various pathophysiological conditions. After 48 h of incubation with the compound, 100 µL of the AGS cell suspension was collected, and the CAT activity was determined according to the protocol of Aebi et al. [38].

### 2.16. Membrane Integrity

#### Determination of Lactate Dehydrogenase (LDH)

One of the most widely used methods to evaluate cytotoxicity in cells is the determination of LDH enzyme activity, which is based on measuring the activity of cytoplasmic enzymes released from damaged cells. After incubation, 100 µL of the cell culture supernatant was collected, and LDH levels were determined following the protocol of Decker et al. [39].

## 3. Results

### 3.1. Synthesis and Integrated Computational Analysis

#### 3.1.1. Synthesis of the Compound

**Figure 2:**
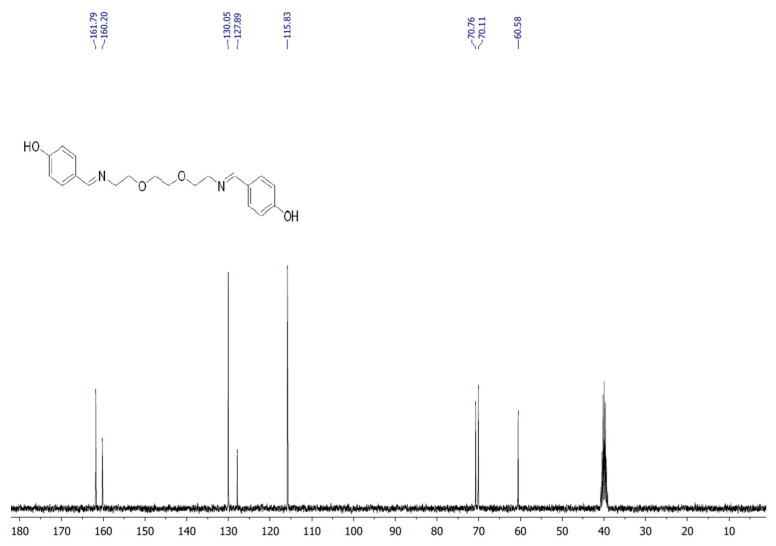
^13^C NMR spectrum of targeted compound.

**Figure 3:**
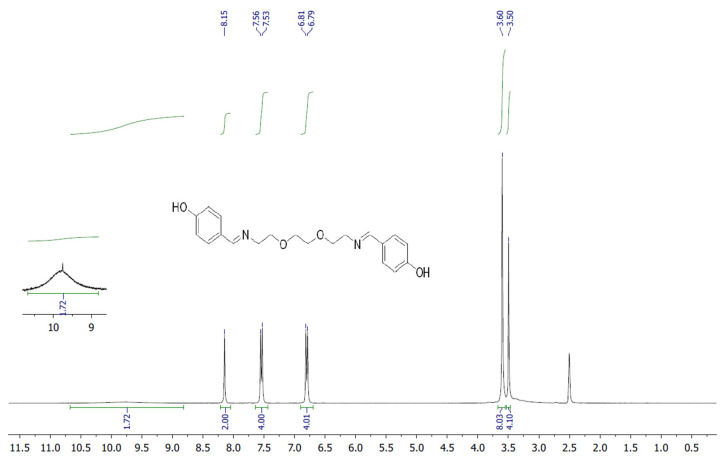
^1^H NMR spectrum of targeted compound.

The target heterocyclic compound, 4,4′-(5,8-dioxa-2,11-diazadodecane-1,11-diene-1,12-diyl)diphenol, was successfully synthesized through a condensation reaction between 4-hydroxybenzaldehyde and 2,2′-(ethane-1,2-diylbis(oxy))bis(ethan-1-amine). This reaction occurs through the formation of an imine bond between the aromatic aldehyde group and the primary amine group. The reaction afforded a white precipitate, which was isolated in good yield (85–90%) with a melting point of 135–136 °C. Structural confirmation was carried out using ^1^H and ^13^C NMR spectroscopy. The ^1^H NMR spectrum (DMSO-d_6_, δ, ppm) exhibited signals at δ 3.5 s (4H, 2CH_2_N), 3.6 s (8H, 4CH_2_O), 6.79–6.81 d (4H, Ar), 7.53–7.56 d (4H, Ar), 8.15 s (2H, 2CH=N), and 9.75 s (2H, 2OH). The ^13^C NMR spectrum (DMSO-d_6_, δ, ppm) showed characteristic resonances at δ 60.58 (2CH_2_N), 70.11 (2CH_2_O), 70.76 (2CH_2_O), 115.83 (2CH_Ar_), 127.89 (2C_Ar_), 130.05 (2CH_Ar_), 160.2 (2C_Ar_), and 161.79 (2CH=N). The disappearance of the aldehyde proton signals and the appearance of the azomethine (CH=N) peaks confirmed the successful formation of the desired compound (Figure 1). Figure 2 and Figure 3 display the ^1^H-NMR spectra of the compounds before and after synthesis, respectively, forming the basis of structural confirmation. In Figure 2, the aromatic and aliphatic proton signals of the starting material are clearly observed. In Figure 3, the characteristic proton signal of the imine group appears at approximately 8.4–8.6 ppm, confirming Schiff base formation. Additionally, the multiplets corresponding to ethylene groups and the signals of aromatic protons support the structural integrity of the molecule. These analyses confirm that the synthesized compound possesses the expected chemical structure (Figure 1).

##### Molecular Docking Analysis

**Figure 4:**
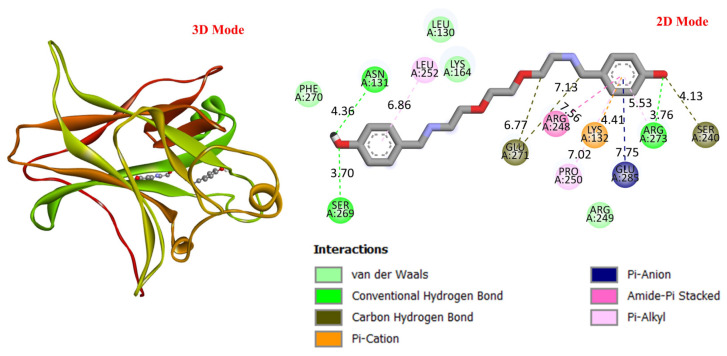
Three-dimensional and two-dimensional images of DODP compound and TP53 (PDB: 1TUP) protein.

**Figure 5:**
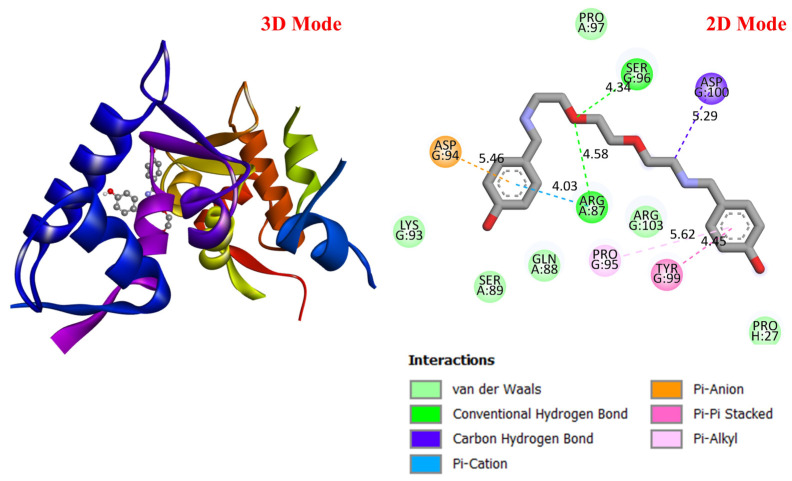
Three-dimensional and two-dimensional images of DODP compound and MDM2 (PDB: 3DAP) protein.

**Figure 6:**
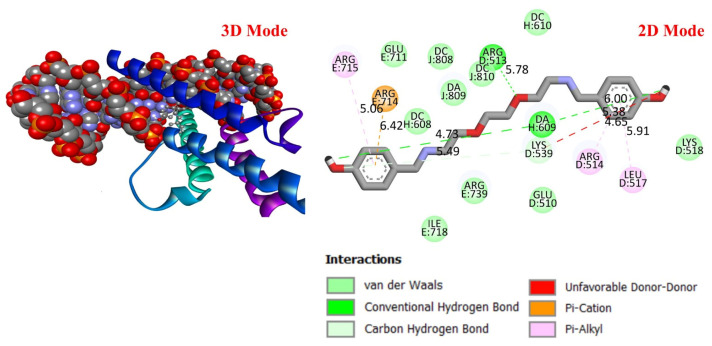
Three-dimensional and two-dimensional images of DODP compound and MYC (PDB: 1NKP) protein.

**Figure 7:**
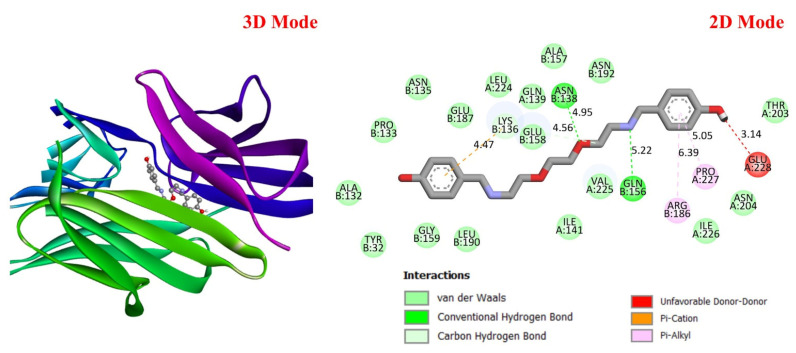
Three-dimensional and two-dimensional images of DODP compound and PD-L1 (PDB: 3BIS) protein.

GC is a leading cause of cancer-related deaths worldwide, and various genetic and molecular mechanisms play a role in its development [40]. In this context, proteins such as TP53, MDM2, MYC, and PD-L1 (CD274) stand out as critical biological targets in the development and progression of GC. TP53 is a tumor suppressor gene that arrests the cell cycle and induces apoptosis in response to DNA damage; however, mutations in this gene are highly prevalent in GC and are associated with treatment resistance [41]. MDM2 is an E3 ligase that negatively regulates TP53, and its overexpression leads to TP53 suppression, contributing to tumorigenesis [42]. Meanwhile, overexpression of the MYC proto-oncogene has been associated with cell proliferation and metastasis and is considered a prognostic biomarker in GC [43]. PD-L1 (CD274) is a checkpoint protein that plays a key role in tumor cell evasion of the immune response and has become a target for immunotherapeutic approaches [44]. In this study, considering the molecular mechanisms of the target proteins in relation to GC, the potential interactions of DODP, a novel synthesized compound, with these proteins were evaluated using in silico molecular docking analyses. This approach aimed to explore the binding affinity and potential inhibitory properties of DODP for the target proteins, providing preliminary insight into its possible therapeutic relevance for GC. Figure 4, Figure 5, Figure 6 and Figure 7 illustrate the interactions between DODP and the studied proteins in both three-dimensional (3D) and two-dimensional (2D) models, which help visualize potential binding modes and interacting amino acids. Table 1 presents the molecular binding interaction scores of DODP and irinotecan molecules with TP53, MYC, MDM2, and PD-L1 (CD274). The strongest interaction was observed with TP53 (−7.80 kcal/mol), followed by PD-L1 (−7.70 kcal/mol), MYC (−7.50 kcal/mol), and MDM2 (−7.30 kcal/mol).

These results suggest that DODP may exhibit relatively higher affinity toward TP53 and PD-L1. Table 2 details the diversity of predicted interactions between DODP and the 1TUP and 3DAP enzymes, including bond lengths. The data indicate that DODP can potentially form hydrogen bonds and pi-based interactions with these enzymes, which may contribute to binding stability. Pi–pi stacking with TYR-99 in 3DAP and hydrogen bonding with ARG-87 are observed in the docking models, which may support potential inhibitory effects. These interactions suggest that DODP may potentially interact with GC-associated enzymes. Table 3 shows the predicted molecular interactions of DODP with the 1NKP and 3BIS enzymes, which may play roles in GC-related pathways such as proliferation, apoptosis, and signal transduction. Hydrogen bonds (4.73–6.00 Å) with ARG-513 and DA-609 in 1NKP, and (4.95–5.22 Å) with GLN-156 and ASN-138 in 3BIS, indicate potential specific binding sites. Additional pi–cation (ARG-714 and LYS-136) and pi–alkyl (ARG-514, LEU-517, and ARG-715) interactions further suggest that DODP may interact with enzyme active sites with moderate-to-high affinity. These docking results suggest that DODP may potentially interact with these enzymes. The molecular docking simulations, presented in both 3D and 2D formats, illustrate potential interactions with the active sites of target proteins. In the 2D models, binding motifs such as hydrogen bonds, π–π stacking, van der Waals forces, and carbon–hydrogen bonds are detailed. These predicted interactions highlight possible stabilizing effects, with lower binding energies suggesting a higher theoretical binding potential (Figure 4, Figure 5, Figure 6 and Figure 7).

**Figure 8:**
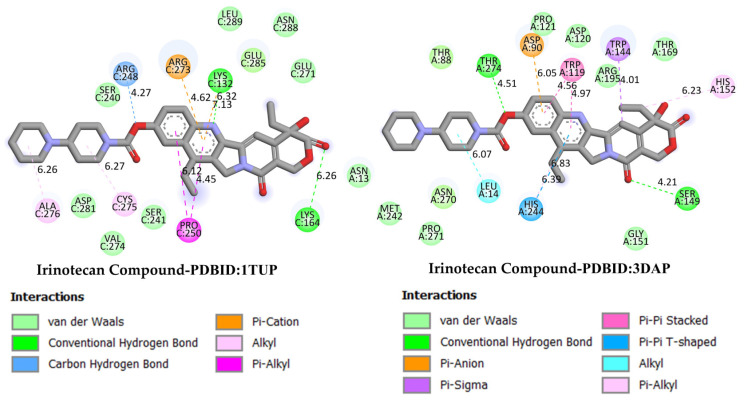
Two-dimensional images of irinotecan compound and TP53 (PDB: 1TUP) and MDM2 (PDB: 3DAP) proteins.

**Figure 9:**
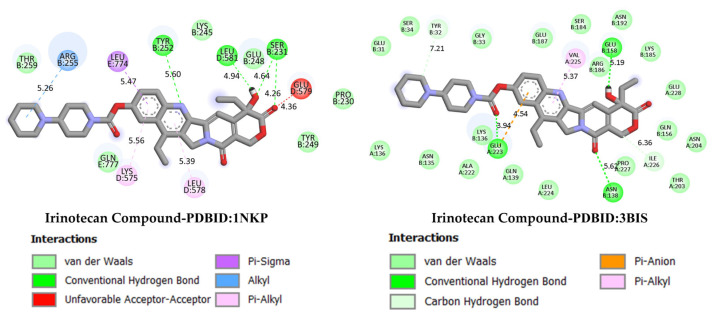
Two-dimensional images of irinotecan compound and MYC (PDB: 1NKP) and PD-L1 (PDB: 3BIS) proteins.

GC is one of the leading causes of cancer-related mortality worldwide, and systemic treatment is the primary approach in advanced-stage cases. Irinotecan, a topoisomerase I inhibitor developed for colorectal cancer, has recently been investigated in various combination regimens due to its potential efficacy in GC treatment. The more negative the docking binding scores, the stronger the interaction of the molecule being studied with the selected biological system. Table 1 also presents the docking score values reflecting the interaction of the irinodecan molecule with the studied biological systems. Molecular docking analyses compared irinotecan as a control ligand and its binding affinities with DODP and the target proteins TP53, MYC, and PD-L1. The findings revealed that DODP exhibited acceptable binding scores for all targets, while the control compound, irinotecan, exhibited stronger interaction profiles, particularly against MYC and PD-L1. Indeed, irinotecan significantly outperformed the MYC protein’s 3DAP and 1NKP structures, with binding energies of −9.30 and −8.70 kcal/mol, respectively, while DODP’s binding energies for the same targets were weaker at −7.50 and −7.30 kcal/mol. Similarly, for PD-L1 (3BIS), irinotecan exhibited a higher affinity than DODP (−7.70 kcal/mol), with a binding score of −8.60 kcal/mol. Although the difference between the two compounds was more limited for the TP53 (1TUP) target (irinotecan: −8.00; DODP: −7.80 kcal/mol), the overall trend supports the stronger interaction with the control ligand. The two-dimensional interaction maps presented in Figure 8 and Figure 9 show that irinotecan forms various van der Waals contacts, hydrogen bonds, and π interactions in the binding sites of TP53, MDM2, MYC, and PD-L1 proteins, confirming, at the structural level, that the compound forms significant and stable complexes with these targets. According to the comparative docking score values given in Table 1, although the new molecule we synthesized did not interact as strongly with the studied proteins as our reference substance, irinodecan, it is seen that the interaction of our new molecule with these biological systems is also significant. This situation reflects that the studied molecule or its derivatives that will be synthesized in the future can be considered in the development of effective drugs for GC.

Finally, the physicochemical and pharmacokinetic properties of DODP were evaluated. Parameters such as Lipinski’s rule compliance, logP/logS values, number of hydrogen bond donors and acceptors (HBD/HBA), topological polar surface area (TPSA), and number of rotatable bonds mostly fall within recommended ranges, suggesting that the compound may have favorable bioavailability. Taken together, these in silico findings indicate that DODP can be considered as a candidate for further research.

##### ADMET Analysis

**Figure 10:**
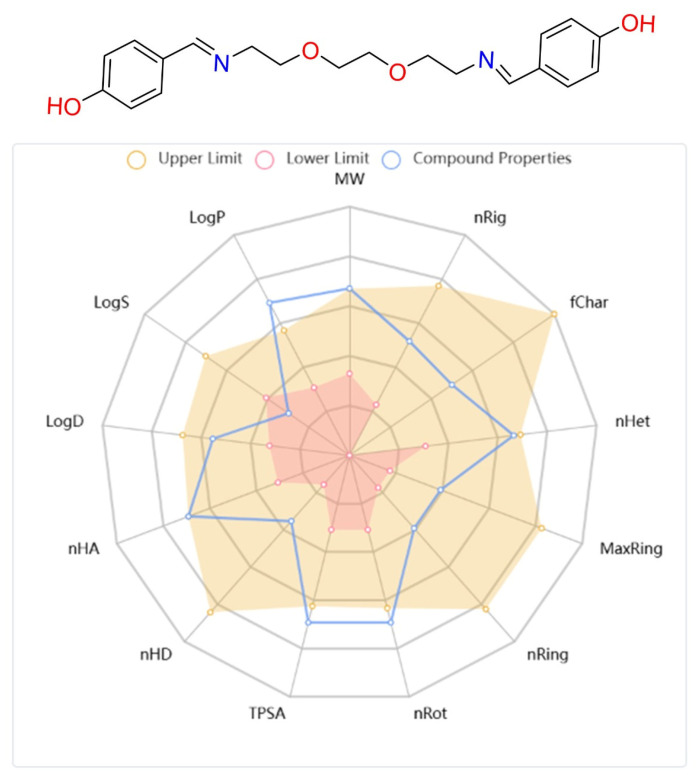
Structural, physicochemical, and lipophilicity parameter images of the DODP compound.

In drug research and development processes, not only the interaction capacity of candidate molecules with target biomolecules but also their pharmacokinetic and toxicological properties are of vital importance for clinical success [45,46,47]. In this context, the ADMET (absorption, distribution, metabolism, elimination, and toxicity) profile is one of the primary parameters determining the success of a compound in the drug development process [48]. In this study, the physicochemical characteristics and ADMET parameters of the synthesized DODP compound were comprehensively evaluated, and based on the obtained data, the pharmacokinetic and pharmacodynamic properties of the compound were revealed in detail (Table 4). In addition, the chemical structure, structural properties, physicochemical parameters, and lipophilicity values of the molecule are visually presented in Figure 10. When the physicochemical, lipophilicity, and ADMET parameters of the DODP molecule were evaluated, it conformed to the Lipinski rule because its molecular weight was 356.17, which was less than 500. Furthermore, the number of hydrogen-bonding acceptors (6) and donors (2) in the molecule was below 12 and 7, respectively, which is consistent with the Lipinski criteria. The LogP value was measured as 1.448, which remained in the range of 0–5, demonstrating that the molecule has a balanced structure in terms of lipophilicity. The topological polar surface area (TPSA) was 83.64 Å^2^, indicating that the molecule has sufficient polarity, suggesting that it is suitable for oral bioavailability. Lipinski’s rules stipulate that these parameters should be within certain limits for molecules to have good oral bioavailability [49,50], and DODP demonstrates this suitability. However, the pharmacokinetic evaluation results indicate that DODP is relatively poorly absorbed from the human intestine and may not maintain effective plasma concentrations for extended periods due to its short half-life. Furthermore, its high CYP3A4 inhibitory activity may increase the risk of potential drug–drug interactions. These limitations can be addressed through further studies to enhance the molecule’s clinical potential. Similarly, Caco-2 cell permeability was determined to be −5.259 log units, which is slightly below the optimal limit, thus offering limited potential for intestinal permeability. The probability of crossing the blood–brain barrier (BBB = 0.00) is also quite low, suggesting that the molecule may be limited in reaching its central nervous system targets. Plasma protein binding is approximately 83.5%, a high but acceptable level that may affect the therapeutic effect of the free drug. The volume of distribution (VD) was determined to be 0.37 L/kg, indicating that the molecule is moderately distributed in body tissues. In terms of metabolic interactions, CYP2D6 inhibitory activity was estimated to be low (0.059), while CYP3A4 inhibitory activity was estimated to be high (0.951), indicating that the molecule carries a risk of interactions, particularly in CYP3A4-mediated drug metabolism. The half-life was 0.974 h, indicating that the molecule has an ultra-short half-life and may require frequent dosing in clinical use. Regarding safety parameters, hERG channel inhibitory activity was estimated to be moderate (0.304), which may indicate a risk of cardiotoxicity. The human liver toxicity (H-HT) and Ames test positivity probabilities were 0.519 and 0.55, respectively, suggesting that the potential for hepatotoxicity and mutagenicity requires careful evaluation. Finally, the risk of acute oral toxicity in mice was estimated to be low (0.129).

#### 3.1.2. Chemical Reactivity

**Figure 11:**
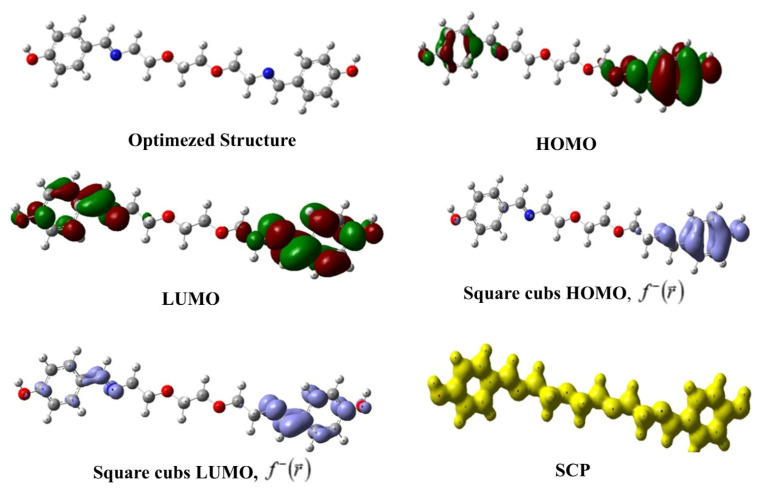
Optimized structure, HOMO, LUMO, square cubs HOMO, square cubs LUMO, and SCP images of the studied compound.

In Table 5, the calculated global reactivity indices for the synthesized compound are presented, while Figure 11 visually gives the optimized structure, HOMO, LUMO, square cubs HOMO, square cubs LUMO, and SCP images of the compound. One of the most popular reactivity descriptors of CDFT is the chemical hardness that became popular with the introduction of the hard and soft acid–base (HSAB) principle to science [51,52,53]. The powerful relation between this quantity and chemical stability is explained through the maximum hardness principle (MHP) of Pearson [54]. According to MHP, chemical systems behave in ways that maximize hardness. This implies that the state where hardness is maximized is lower in energy and more stable. Another electronic structure principle used in global reactivity analysis is the minimum electrophilicity principle (MEP) [55]. The MEP states that electrophilicity is a minimized property, such as polarizability, in stable states. The values presented in the relevant table indicate that the synthesized molecule has relatively high chemical hardness and low electrophilicity index and, thus, can exhibit high stability.

#### 3.1.3. Bioinformatics Analysis

**Figure 12:**
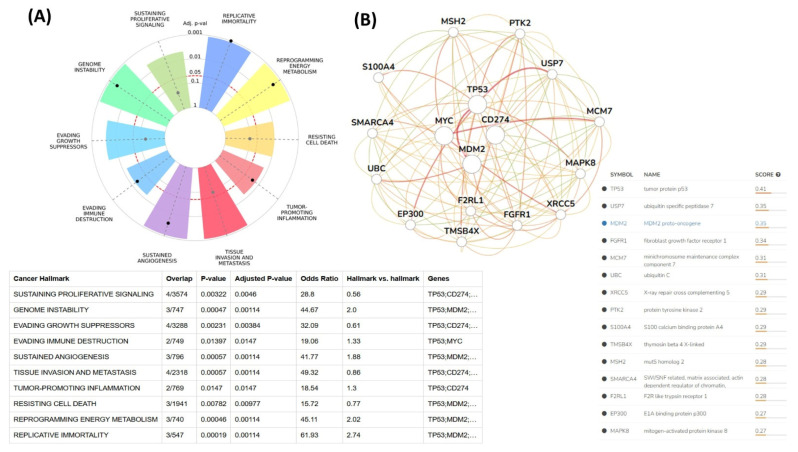
(**A**) Cancer hallmark enrichment analysis. (**B**) PPI network analysis of *CD274* and associated genes.

**Figure 13:**
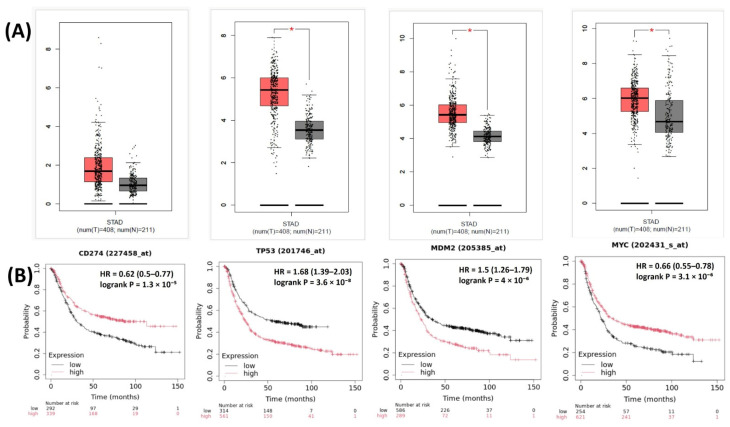
(**A**) Expression levels of *CD274*, *TP53*, *MDM2*, and *MYC* in STAD tumor vs. normal tissues. (**B**) Overall survival analysis based on high- vs. low-expression groups for the same genes in TCGA-STAD. * Significantly different expression levels were observed between tumor and normal tissues (*p* < 0.05).

**Figure 14:**
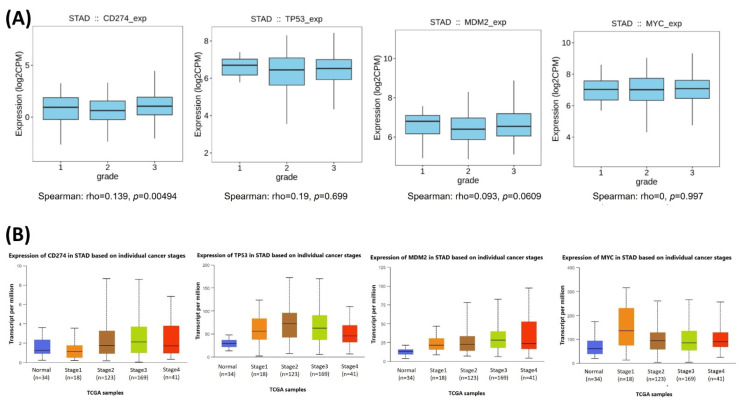
(**A**) Boxplot visualization of *CD274*, *TP53*, *MDM2*, and *MYC* expression levels across tumor grades (grades 1–3) in TCGA-STAD. (**B**) Expression distribution of the same genes across individual cancer stages.

**Figure 15:**
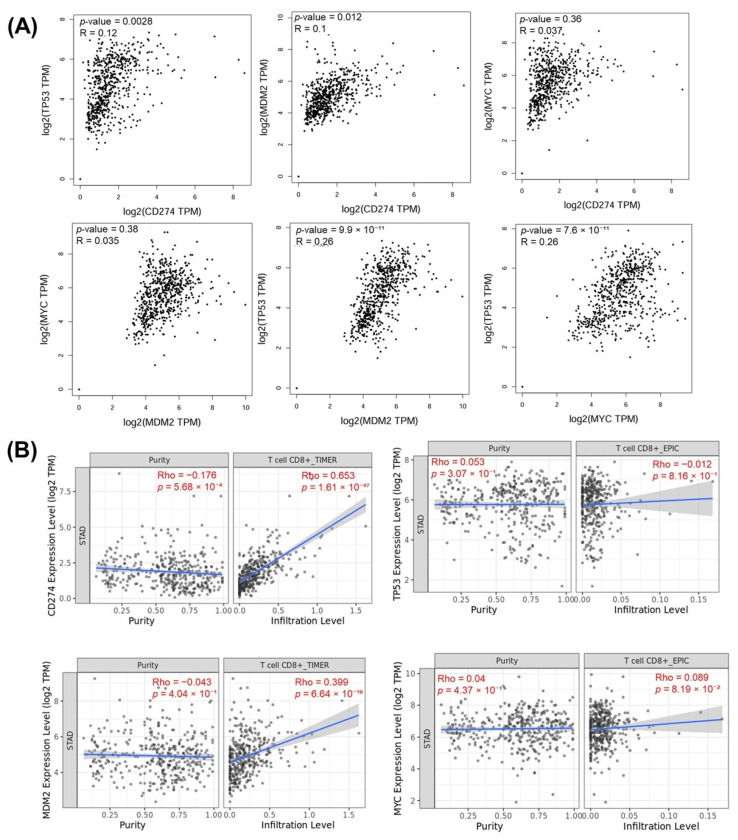
(**A**) Gene-to-gene correlation analysis showing the relationships among *CD274*, *TP53*, *MDM2*, and *MYC* expression levels in TCGA-STAD samples. (**B**) Correlation with immune purity and CD8+ T-cell infiltration.

**Figure 16:**
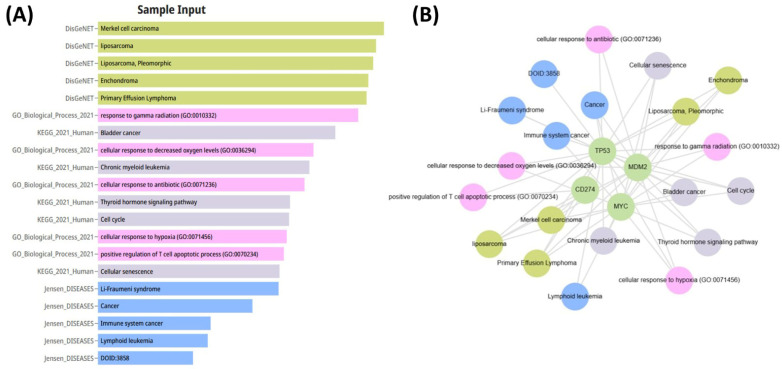
(**A**) Functional enrichment analysis performed using DisGeNET, GO, and KEGG databases, showing significantly enriched pathways and biological processes related to tumor biology. (**B**) Gene–disease association network illustrating the links between *CD274*, *TP53*, *MDM2*, and *MYC* and their associated diseases and functional pathways.

**Figure 17:**
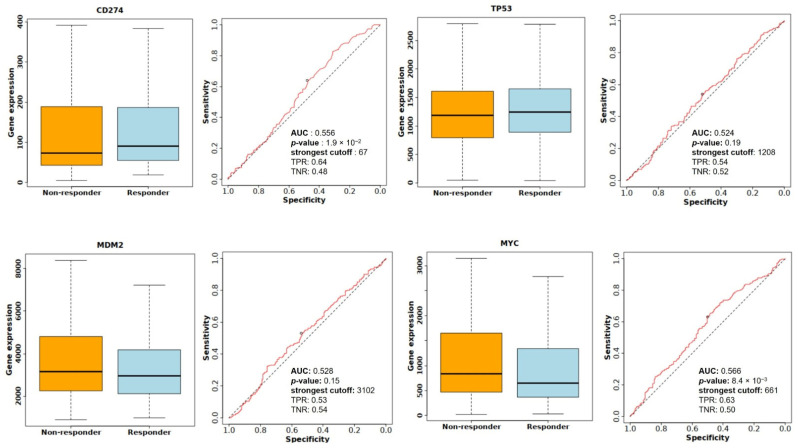
Comparison of *CD274*, *TP53*, *MDM2*, and *MYC* expression levels between responder and non-responder groups, along with ROC analysis evaluating predictive performance for treatment response.

**Figure 18:**
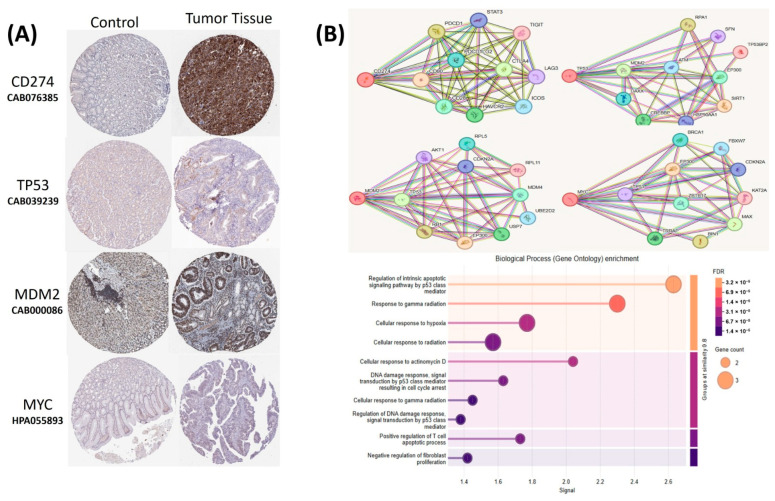
(**A**) ImmuRepresentative immunohistochemical staining images of *CD274*, *TP53*, *MDM2*, and *MYC* in normal and tumor gastric tissues obtained from the Human Protein Atlas (HPA) database. The images are provided by the HPA repository and are shown as originally presented. (**B**) Protein–protein interaction (PPI) network and Gene Ontology (GO) enrichment analysis of *CD274*, *TP53*, *MDM2*, and *MYC* based on STRING database results.

The relationships between the investigated genes and the ten hallmarks of cancer were evaluated using gene set enrichment analysis. Significant enrichment was observed particularly for “replicative immortality”, “tissue invasion and metastasis”, and “genome instability”, indicating associations with these processes (Figure 12A). PPI network analysis showed that *TP53*, *MDM2*, *MYC*, and *CD274* were positioned centrally within interaction networks, reflecting their involvement in multiple signaling pathways (Figure 12B). TCGA analyses demonstrated higher expression of *CD274*, *TP53*, *MDM2*, and *MYC* in tumor tissues compared with normal tissues (Figure 13A,B). Correlation analyses in gastric adenocarcinoma samples revealed significant interactions among *CD274*, *TP53*, *MYC*, and *MDM2* (Figure 14A,B). Immune infiltration assessments showed positive associations between *CD274*/*MDM2* and CD8+ T-cell infiltration, while *TP53* and *MYC* exhibited negative correlations (Figure 15). Among these, only *CD274* expression showed a statistically significant increase with tumor grade. No significant differences were detected across tumor stages. Functional enrichment analyses highlighted involvement of the studied genes in pathways such as cell cycle regulation, hypoxia response, and immune system processes (Figure 16A,B). ROC curve evaluation indicated that *MYC* and *MDM2* displayed measurable distinction between responders and non-responders (Figure 17). Immunohistochemistry data confirmed increased expression of *CD274* and *TP53* in tumor tissues. STRING-based analysis supported a strong interaction between *TP53* and *MDM2*, particularly in pathways associated with DNA damage response and apoptosis regulation (Figure 18A,B).

### 3.2. Experimental Findings

#### 3.2.1. Demographic and Clinical Characteristics

A total of 60 participants were included in this study, comprising 30 patients with GC and 30 healthy controls (Table 6). The mean ages of the control and GC groups were 59.03 ± 9.90 and 60.43 ± 10.80 years, respectively, with no statistically significant difference between the two groups (*p* = 0.603). The gender distribution was similar across groups (*p* = 0.688). However, a significant difference was observed in the smoking history (*p* = 0.001), with a higher proportion of smokers in the control group compared with GC patients. Alcohol consumption was significantly more frequent among GC patients (*p* = 0.018). No significant difference was detected in the family history of GC between the groups (gene) (Table 6). Smoking, alcohol consumption, and cancer history significantly contributed to group differences after multivariate adjustment (adjusted R^2^ = 0.239; *p* < 0.001). Smoking demonstrated the strongest association (β = −0.487; *p* < 0.001), followed by cancer history (β = −0.246; *p* = 0.044), while alcohol showed a non-significant trend toward group separation (β = 0.192; *p* = 0.112) (Table 7). These findings indicate that smoking and cancer history act as important confounders and should be considered when interpreting oxidative stress and gene expression results, whereas the potential role of alcohol may require further evaluation in larger cohorts.

**Figure 19:**
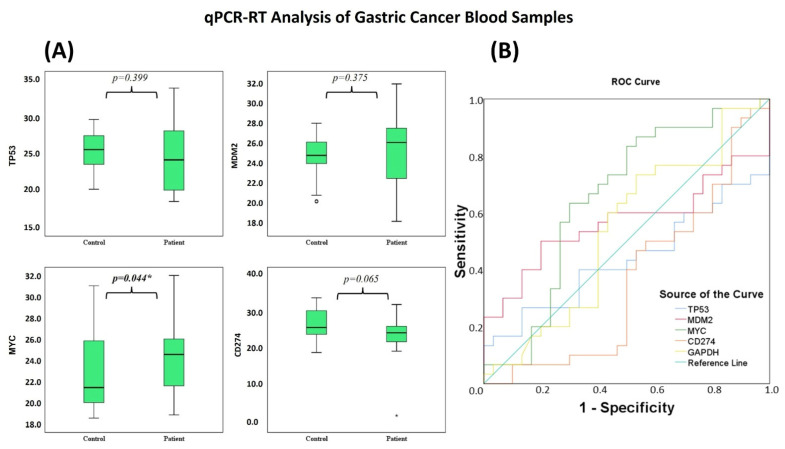
(**A**) Boxplots display expression differences of *TP53*, *MDM2*, *MYC*, and *CD274* between GC patients and controls. Significant differences in expression levels were observed between the patient and control groups (* *p* < 0.05). (**B**) ROC curves show the diagnostic performance of these genes in distinguishing GC from controls.

In contrast with the significant transcriptional modulation observed in AGS cells, gene expression differences in peripheral blood samples were more modest. qPCR results showed no statistically significant differences in the *TP53*, *MDM2*, and *CD274* genes in peripheral blood (*p* > 0.05) (Table 8). In contrast, *MYC* was the only gene that showed a statistically significant difference between groups and showed low discriminatory ability in the ROC analysis (AUC = 0.651; *p* = 0.044) (Table 9 and Table 10; Figure 19A,B).

#### 3.2.2. In Vitro Cytotoxicity Assay (MTT Assay)

**Figure 20:**
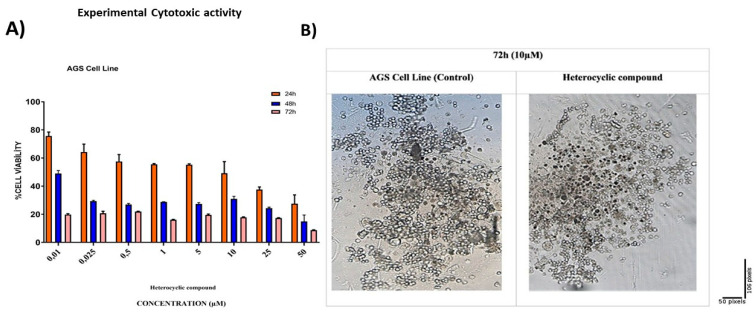
(**A**) The dose- and time-dependent cytotoxic effects of the heterocyclic compound on AGS GC cells were measured at 24, 48, and 72 h using varying concentrations. (**B**) The administration of 10 µM of the compound resulted in the disruption of the membrane integrity of the AGS cells compared with the control group. The administration of 10 µM of the compound resulted in the disruption of the membrane integrity of the AGS cells compared with the control group. Effects of DODP treatment on the morphological changes of AGS cells observed under an inverted microscope (Scale bar = 50 µM)

The synthesized compound was applied to AGS cells at concentrations ranging from 0.01 to 50 µM. The IC_50_ values of the heterocyclic compound for AGS cells were 21.56 µM at 24 h, 8.36 µM at 48 h, and 0.73 µM at 72 h. The IC_50_ value was calculated with the GraphPad Prism 7 software (Figure 20A, Table 11).

#### 3.2.3. Cell Morphology Analysis

Following exposure to the compound, AGS cells were examined for morphological changes. After incubation, pronounced morphological alterations were observed at the 10 µM concentration (Figure 20B).

#### 3.2.4. qPCR Results of Drug-Treated AGS Cell Lysates

For qPCR, total RNA was isolated from AGS cell lysates treated with the compound at 10 µM for 48 h, matching the conditions used in the antioxidant assays and ensuring sub-cytotoxic viability levels.

#### 3.2.5. Differential Cellular Expressions of *TP53*, *MDM2*, *MYC*, and *PD-L1* Genes in GC Samples

**Figure 21:**
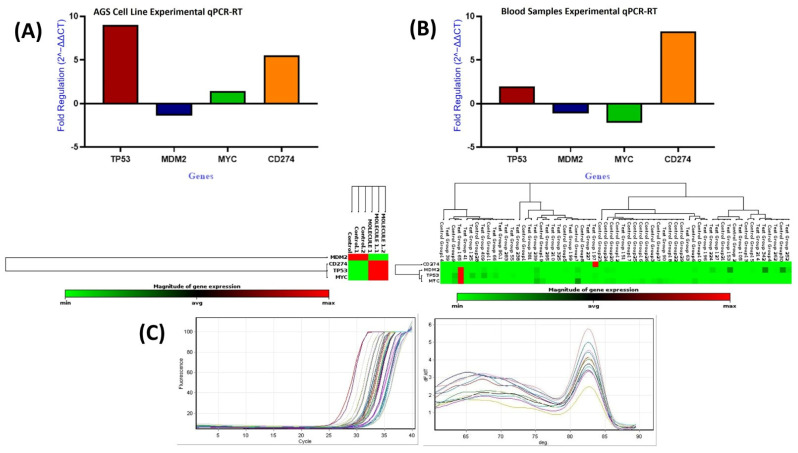
Relative mRNA expression of *TP53*, *MDM2*, *MYC*, and *CD274* in AGS cells (**A**) and blood samples (**B**), and qPCR amplification and melt-curve plots (**C**).

The data were analyzed using Student’s *t*-test to compare mean gene expression levels between the treated and control groups (Figure 21A; Table 12). qPCR analysis showed significant transcriptional changes following compound treatment. *PD-L1* expression increased by approximately 5.5-fold (*p* < 0.0001), indicating a marked stress-associated upregulation of an immune-related gene. *TP53* exhibited a nine-fold increase (*p* < 0.0001), consistent with activation of DNA damage response pathways. *MYC* showed a modest but statistically significant increase (1.42-fold; *p* < 0.0001). In contrast, *MDM2*, a known negative regulator of *TP53*, was significantly downregulated (−1.38-fold; *p* < 0.0001). The stability of *GAPDH* expression confirmed the reliability of the normalization strategy.

#### 3.2.6. Determination of Antioxidant Levels

**Figure 22:**
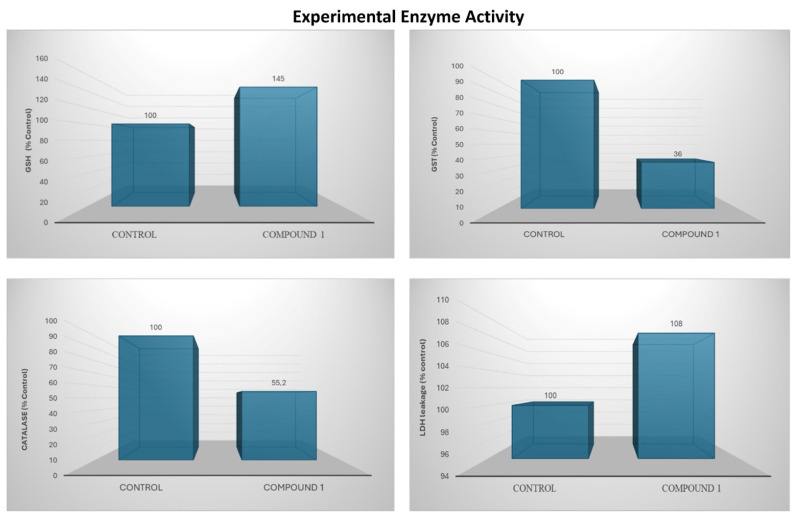
Bar charts illustrate alterations in oxidative stress markers and antioxidant enzyme activity following compound treatment.

For the antioxidant assays, AGS cells were treated with the optimal concentration of 10 µM, determined from the 48h IC_50_-based evaluation of cell lysates. This dose was selected to maintain cell viability above 70% and to avoid interference from cell death. Under these conditions, GSH levels increased by 45% compared with the control group. In contrast, GST and CAT activities decreased by 64% and 45%, respectively. LDH leakage increased by 8%, indicating a mild early cytotoxic response without substantial membrane damage (Figure 22).

## 4. Discussion

Recent studies have shown that azomethine derivatives may influence important cancer-related pathways, including *PD-L1*, *TP53*, and *MYC* in gastric tumors [56,57,58,59]. In this study, DODP (Figure 1) demonstrated a time-dependent cytotoxic effect in AGS GC cells and modulated the expression of *PD-L1*, *TP53*, *MDM2*, and *MYC*, which are involved in tumor growth control and immune escape (Figure 21; Table 12). The significant upregulation of *TP53* and reduction in *MDM2* suggest that DODP may influence apoptotic signaling, whereas the modest increase in *MYC* expression indicates limited sensitivity within proliferative pathways (Figure 21A). The increase in *PD-L1* expression observed in AGS cells is likely a stress-related response rather than a mechanism of immune evasion [60,61,62,63]. *TP53* expression showed a significant increase after DODP treatment. Since *TP53* is a key regulator of DNA damage response and apoptosis [64,65,66], this transcriptional shift may indicate activation of tumor suppressor-associated pathways. Meanwhile, the reduction in *MDM2*, a primary negative regulator of *TP53* [42], is consistent with this response, although confirmation at the protein level will be required to support this interpretation. *MYC*, an important regulator of proliferation and metabolism [65,66], exhibited a statistically significant yet modest increase, suggesting that its sensitivity to DODP is more limited compared with *TP53*. These findings collectively indicate that DODP affects genes associated with cell survival and proliferation control. In addition, the oxidative stress profile indicates a controlled cytotoxic response (Figure 22). Rather than the typical depletion of GSH observed with strong prooxidant agents, the cells exhibited a transient GSH increase accompanied by a marked decrease in GST and CAT activities, suggesting an early compensatory defense that progressively weakens. This redox imbalance is mechanistically aligned with the observed transcriptional changes. The shift toward reduced detoxification capacity favors apoptotic signaling, consistent with the significant upregulation of *TP53* and downregulation of its negative regulator *MDM2*, indicating a p53-dependent stress response. Likewise, *PD-L1* elevation likely reflects a stress-induced transcriptional mechanism rather than immune-evasive behavior. Together, these findings suggest that DODP induces a moderate, apoptosis-oriented oxidative stress that contributes to the regulation of key tumor-related genes. Although DODP markedly altered the expression of *TP53*, *MDM2*, *PD-L1*, and *MYC* in AGS cells, these transcriptional changes were not fully reflected in the systemic circulation of GC patients (Figure 19A; Table 12). In particular, *TP53*, *MDM2,* and *PD-L1* showed no statistically significant differences in peripheral blood (*p* > 0.05) (Table 9), indicating that the strong in vitro effects cannot be interpreted as clinically relevant biomarker alterations at this stage. Only *MYC* exhibited a modest but statistically significant discriminatory signal in ROC analysis (Table 9; Figure 19B), which suggests limited diagnostic sensitivity but does not imply clinical utility. This difference likely reflects that tumor-specific gene changes are diluted and more tightly regulated when measured in blood. Bioinformatics analyses showed that *TP53*, *MDM2*, *MYC*, and *PD-L1* are involved in cancer-related regulatory pathways and are predicted to interact within shared molecular networks. Gene enrichment indicated their participation in processes such as cell cycle control, stress response, and apoptosis. The immunohistochemical data confirm elevated expression of these proteins in gastric tumor tissues, supporting their biological relevance in GC.

Therefore, based on the current results, DODP-mediated gene regulation should be considered as preliminary mechanistic evidence, and no biomarker level conclusions can yet be drawn without validation in tumor tissue or larger patient cohorts.

## 5. Conclusions

DODP exerted a controlled cytotoxic effect in AGS cells by modulating genes associated with apoptosis and stress response. However, the limited gene expression changes observed in patient blood samples indicate that these molecular effects require further validation at the systemic level. Overall, the findings provide preliminary mechanistic insight, and additional in vivo studies are needed to better clarify the biological impact of DODP.

## 6. Limitations

This study had several limitations. L929 fibroblasts could not be reliably evaluated due to loss of viability, and the absence of in vivo experiments limits the assessment of therapeutic relevance. Flow cytometry and Western blot analyses were not performed because of financial and technical constraints and should be included in future studies.

## Figures and Tables

**Table 1:** Molecular docking interaction scores with TP53, MDM2, MYC, and PD-L1 (CD274) proteins of DODP and irinotecan compounds.

Compound	TP53 (PDB: 1TUP)	MDM2 (PDB: 3DAP)	MYC (PDB: 1NKP)	PD-L1 (PDB: 3BIS)
DODP	−7.80	−7.50	−7.30	−7.70
Irinotecan	−8.00	−9.30	−8.70	−8.60

**Table 2:** The interaction parameters between DODP compound and 1TUP and 3DAP enzymes.

Type of Bond	Interacting Amino Acid (PDB: 1TUP)	Bond Length (Å)	Interacting Amino Acid (PDB: 3DAP)	Bond Length (Å)
Van der Waals interactions	PHE-270 LEU-130 ARG-249 LYS-164	-	LYS-93 SER-89 GLN-88 ARG-103	-
Conventional hydrogen bonds	ASN-131 ARG-273 SER-269	4.36 3.76 3.70	ARG-87 SER-96	4.58 4.34
Carbon–hydrogen bond	GLU-271 GLU-271 SER-240	6.77 7.13 4.13	ASP-100	5.29
Pi–cation	LYS-132	4.41	ASP-94	5.46
Pi–anion	GLU-285	7.75	-	-
Amide–Pi stacked	ARG-248	7.56	-	-
Pi–Alkyl	LEU-252 PRO-250	6.86 7.02	PRO-95	5.62
Pi–Pi stacked	-	-	TYR-99	4.45

**Table 3:** The interaction parameters between DODP compound and 1NKP and 3BIS enzymes.

Type of Bond	Interacting Amino Acid (PDB: 1NKP)	Bond Length (Å)	Interacting Amino Acid (PDB: 3BIS)	Bond Length (Å)
Van der Waals interactions	GLU-711 LYS-518 GLU-510 ARG-739 ILE-718	-	THR-203 ASN-192 ALA-157 LEU-224 PRO-133 GLY-159 LEU-190	-
Conventional hydrogen bonds	DA-609 ARG-513 DA-609	4.73 5.78 6.00	GLN-156 ASN-138	5.22 4.95
Carbon–hydrogen bond	LYS-539	5.49	GLU-158	4.56
Unfavorable donor–donor	LYS-539	5.38	GLU-228	3.14
Pi–cation	ARG-714	6.42	LYS-136	4.47
Pi–Alkyl	ARG-514 LEU-517 ARG-715	4.65 5.91 5.06	ARG-186 PRO-227	6.39 5.05

**Table 4:** DODP molecule’s physicochemical, lipophilicity, and ADMET parameters.

Property	DODP	Comment
Molecular weight	356.17	Molecular weight < 500
nHA	6	Hydrogen bond acceptors < 12
nHD	2	Hydrogen bond donors < 7
logP	1.448	Log of the octanol/water partition coefficient: 0–5
TPSA	83.64	Topological polar surface area: 0–140
HIA	0.00	Category 1: HIA+ (HIA < 30%); Category 0: HIA- (HIA ≥ 30%); The output value is the probability of being HIA+
Caco-2 permeability	−5.259	Optimal: higher than −5.15 Log unit
BBB	0.00	The output value is the probability of being BBB+
PPB	83.536	Optimal: <90%. Drugs with high protein binding may have a low therapeutic index
VD	0.37	Optimal: 0.04–20 L/kg
CYP2D6	0.059	Category 1: inhibitor; Category 0: non-inhibitor The output value is the probability of being an inhibitor
CYP3A4	0.951	Category 1: inhibitor; Category 0: non-inhibitor The output value is the probability of being an inhibitor
T_1/2_	0.974	The unit of predicted T_1/2_ is hours Ultra-short-half-life drugs: T_1/2_ < 1 h; short-half-life drugs: T_1/2_ between 1 and 4 h; intermediate short-half-life drugs: T_1/2_ between 4 and 8 h; and long-half-life drugs: T_1/2_ > 8 h
hERG blockers	0.304	Category 1: active; Category 0: inactive The output value is the probability of being active
H-HT	0.519	Human hepatotoxicity Category 1: H-HT positive (+); Category 0: H-HT negative (−) The output value is the probability of being toxic
AMES toxicity	0.55	Category 1: Ames-positive (+); Category 0: Ames-negative (−) The output value is the probability of being toxic.
Rat oral acute toxicity	0.129	Category 0: low toxicity; Category 1: high toxicity The output value is the probability of being highly toxic

**Table 5:** Calculated global reactivity indices for the synthesized compound.

E_HOMO_ (eV)	−5.932
E_LUMO_ (eV)	−0.956
Chemical potential, µ	−3.444
Electronegativity, χ	3.444
Chemical hardness, η	4.976
Electrophilicity index, ω	1.196

**Table 6:** Demographic data and clinical characteristics of GC patients and healthy controls.

	Control n (%)	GC n (%)	*p*-Value
Number of individuals	30 (100.0)	30 (100.0)	
Gender			*p* = 0.688 ^a^
Male	27 (50.9)	26 (49.1)	
Female	3 (42.9)	4 (57.1)	
Cigarette history			
Smokers			*p* = 0.001 ^a,^*
Male	23 (67.6)	11 (32.4)	
Female	-	-	
Alcohol history			
Drinkers			
Male	1 (12.5)	7 (87.5)	*p* = 0.018 ^a,^*
Female	-	-	
Family history of GC			
Yes			*p* = 0.959 ^a^
Male	3 (50.0)	3 (50.0)	
Female	0 (0.0)	1 (100.0)	
Age			
Range	46–85	40–79	*p* = 0.603 ^b^
Average age	59.03 ± 9.90	60.43 ± 10.80	

Significant indicates * *p* < 0.05; ^a^ chi-square test with n (%), and ^b^ independent *t*-test (mean ± standard deviation).

**Table 7:** Multivariate regression analysis evaluating the influence of smoking and cancer history on group classification.

Variable	B	SE	β	t	*p*-Value	95% CI (Lower–Upper)
Constant	1.795	0.100	—	17.889	<0.001 *	1.594–1.996
Cancer history	−0.298	0.145	−0.246	−2.064	0.044 *	−0.588–0.009
Smoking	−0.491	0.118	−0.487	−4.163	<0.001 *	−0.727–0.255
Alcohol	0.240	0.149	0.192	1.616	0.112	−0.058–0.538

Significant indicates * *p* < 0.05; multivariate linear regression analysis.

**Table 8:** qPCR results showing CT values, fold regulation, and statistical differences between the GC patient group and the control group.

Genes	Groups	Mean CT	Fold Regulation	*p*-Value
*CD274*	Group 1 Control	24.09 26.78	8.28	>0.05 ^a^
*TP53*	Group 1 Control	24.73 25.34	1.95	>0.05 ^a^
*MYC*	Group 1 Control	24.45 22.95	−2.20	>0.05 ^a^
*MDM2*	Group 1 Control	25.20 24.70	−1.10	>0.05 ^a^
*GAPDH*	Group 1 Control	26.53 26.17	1.00	N/A

Significant indicates *p* > 0.05, ^a^ Student’s *t*-test, *GAPDH*: glyceraldehyde 3-phosphate dehydrogenase, and CT: cycle threshold.

**Table 9:** Comparison of gene expression levels between GC patients and healthy controls.

Genes	Control (30)	GC (30)	*p*-Value
*TP53*	25.33 ± 2.62	24.73 ± 4.27	*p* = 0.399 ^a^
*MDM2*	24.69± 2.23	25.19 ± 3.91	*p* = 0.375 ^a^
*MYC*	22.95 ± 3.79	24.44 ± 3.17	*p* = 0.044 * ^,^^a^
*PDL-1 (CD274)*	26.77 ± 4.36	24.08 ± 5.11	*p* = 0.065 ^a^

Significant indicates * *p* < 0.05; ^a^ Mann–Whitney U test (mean ± standard deviation).

**Table 10:** Receiver operating characteristic (ROC) curve analysis of *TP53*, *MDM2*, *MYC*, and *CD274* gene expression for distinguishing GC from controls.

-	*TP53*	*MDM2*	*MYC*	*PDL-1 (CD274)*
AUC (95% CI)	0.437 (0.286–0.587)	0.567 (0.413–0.720)	0.651 (0.507–0.795)	0.361 (0.218–0.504)
*p*-values	0.399	0.375	0.044 *	0.065
Cut-off point	≥24.02	≥24.72	≥22.9	≤24.2
Sensitivity (%)	53.3	60.0	70.0	60.0
Specificity (%)	66.7	60.0	60.0	56.7
PPV (%)	61.5	60.0	63.6	58.5
NPV (%)	58.8	60.0	66.7	58.2

Significant indicates * *p* < 0.05; AUC: area under the ROC curve, CI: confidence interval, PPV: positive predictive value, and NPV: negative predictive value.

**Table 11:** Cytotoxic activity of the heterocyclic compound against AGS cells at different incubation times.

IC_50_ (µM)			
Incubation period	24 h	48 h	72 h
Heterocyclic compound	21.56	8.36	0.73
IC_50_: half-maximal inhibitory concentration			

**Table 12:** qPCR results showing CT values, fold regulation, and statistical differences among experimental groups in the AGS cell line.

Genes	Groups	Mean CT	Fold Regulation	*p*-Value
*CD274*	Group 1 Control	30.41 31.07	5.50	<0.0001 *^,a^
*TP53*	Group 1 Control	29.65 31.02	9.00	<0.0001 *^,a^
*MYC*	Group 1 Control	28.11 26.82	1.42	<0.0001 *^,a^
*MDM2*	Group 1 Control	31.99 29.73	−1.38	<0.0001 *^,a^
*GAPDH*	Group 1 Control	30.96 29.16	1.00	N/A

Significant indicates * *p* < 0.0001; ^a^ Student’s *t*-test, *GAPDH*: glyceraldehyde 3-phosphate dehydrogenase, and CT: cycle threshold.

## Data Availability

The original contributions presented in this study are included in the article. Further inquiries can be directed to the corresponding author.

## Abbreviations

The following abbreviations were used in this manuscript:

ADMET	Absorption, Distribution, Metabolism, Elimination, and Toxicity
AGS	Adenocarcinoma Gastric Cell Line
AUC	Area Under the Curve
BBB	Blood–Brain Barrier
CAT	Catalase
CDFT	Conceptual Density Functional Theory
CI	Confidence Interval
CYP2D6/CYP3A4	Cytochrome P450 Isoenzymes
DFT	Density Functional Theory
DMEM	Dulbecco’s modified Eagle’s medium
FBS	Fetal Bovine Serum
GAPDH	Glyceraldehyde-3-Phosphate Dehydrogenase
GC	Gastric Cancer
GO	Gene Ontology
GSH	Glutathione
GST	Glutathione S-Transferase
H-HT	Human Hepatotoxicity
HIA	Human Intestinal Absorption
HOMO	Highest Occupied Molecular Orbital
KEGG	Kyoto Encyclopedia of Genes and Genomes
LDH	Lactate Dehydrogenase
LUMO	Lowest Unoccupied Molecular Orbital
MDM2	Mouse Double Minute 2 Homolog
MYC	Myelocytomatosis Oncogene
NMR	Nuclear Magnetic Resonance
NPV	Negative Predictive Value
PDB	Protein Data Bank
PD-L1 (CD274)	Programmed Death-Ligand 1
PPB	Plasma Protein Binding
PPI	Protein–Protein Interaction
PPV	Positive Predictive Value
ROC	Receiver Operating Characteristic
ROS	Reactive Oxygen Species
qRT-PCR	Real-Time Quantitative Reverse Transcription PCR
SCP	Square Cubes of Potential (in Molecular Orbital Analysis)
SPSS	Statistical Package for the Social Sciences
T_1/2_	Half-Life
TP53	Tumor Protein p53
VD	Volume of Distribution

## References

[1] Wong M.C.S., Huang J., Chan P.S.F., Choi P., Lao X.Q., Chan S.M., Teoh A., Liang P. (2021). Global incidence and mortality of gastric cancer, 1980–2018. JAMA Netw. Open.

[2] Cummings D., Wong J., Palm R., Hoffe S., Almhanna K., Vignesh S. (2021). Epidemiology, diagnosis, staging and multimodal therapy of esophageal and gastric tumors. Cancers.

[3] Ko K.P. (2023). Risk factors of gastric cancer and lifestyle modification for prevention. J. Gastric Cancer.

[4] Richa, Sharma N., Sageena G. (2022). Dietary factors associated with gastric cancer: A review. Transl. Med. Commun..

[5] Collatuzzo G., Pelucchi C., Negri E., López-Carrillo L., Tsugane S., Hidaka A., Hamada G.S., Hernández-Ramírez R.U., López-Cervantes M., Malekzadeh R. (2021). Exploring the interactions between Helicobacter pylori infection and other risk factors of gastric cancer: A pooled analysis in the StoP project. Int. J. Cancer.

[6] Deng W., Jin L., Zhu H., Vasiliou V., Zhang Y. (2021). Alcohol consumption and risk of stomach cancer: A meta-analysis. Chem. Biol. Interact..

[7] Marques-Lespier J.M., Gonzalez-Pons M., Cruz-Correa M. (2016). Current perspectives on gastric cancer. Gastroenterol. Clin. N. Am..

[8] Lameire N. (2014). Nephrotoxicity of recent anti-cancer agents. Clin. Kidney J..

[9] Makino H. (2004). Treatment and care of neurotoxicity from taxane anticancer agents. Breast Cancer.

[10] Chattaraj A., Syed M.P., Low C.A., Owonikoko T.K. (2023). Cisplatin-induced ototoxicity: A concise review of the burden, prevention, and interception strategies. JCO Oncol. Pract..

[11] Shakdofa M.M., El-Saied F.A., Al-Hakimi A.N. (2012). Synthesis, spectroscopic characterization and biological activity of 2-(p-toluidino)-N′-(2-hydroxbenzylidene) acetohydrazide complexes. Main Group Chem..

[12] Rezayati S., Ramazani A., Sajjafar S., Aghahosseini H., Rezaei A. (2021). Design of a Schiff base complex of copper coated on epoxy-modified core–shell MNPs as an environmentally friendly and novel catalyst for the one-pot synthesis of various chromene-annulated heterocycles. RSC Adv..

[13] Haj N.Q., Mohammed M.O., Mohammood L.E. (2020). Synthesis and biological evaluation of three new chitosan Schiff base derivatives. ACS Omega.

[14] Burlov A.S., Vlasenko V.G., Koshchienko Y.V., Makarova N.I., Zubenko A.A., Drobin Y.D., Fetisov L.N., Kolodina A.A., Zubavichus Y.V., Trigub A.L. (2018). Synthesis, characterization, luminescent properties and biological activities of zinc complexes with bidentate azomethine Schiff-base ligands. Polyhedron.

[15] Matthews H.K., Bertoli C., de Bruin R.A. (2022). Cell cycle control in cancer. Nat. Rev. Mol. Cell Biol..

[16] Singh S.R., Bhaskar R., Ghosh S., Yarlagadda B., Singh K.K., Verma P., Sengupta S., Mladenov M., Hadzi-Petrushev N., Avtanski D. (2025). Exploring the genetic orchestra of cancer: The interplay between oncogenes and tumor-suppressor genes. Cureus.

[17] Reed J.C. (2001). Apoptosis-regulating proteins as targets for drug discovery. Trends Mol. Med..

[18] Narita Y., Muro K. (2023). Updated immunotherapy for gastric cancer. J. Clin. Med..

[19] Stine Z.E., Schug Z.T., Salvino J.M., Dang C.V. (2022). Targeting cancer metabolism in the era of precision oncology. Nat. Rev. Drug Discov..

[20] Mohammed S.A., Kadhim A.J., Salam M. (2023). A comprehensive review of anticancer drug therapy: Advances and challenges. Cancer.

[21] Eslami M., Memarsadeghi O., Davarpanah A., Arti A., Nayernia K., Behnam B. (2024). Overcoming chemotherapy resistance in metastatic cancer: A comprehensive review. Biomedicines.

[22] (2019). Maestro, Schrödinger Release 1.

[23] Burley S.K., Berman H.M., Bhikadiya C., Bi C., Chen L., Di Costanzo L., Christie C., Dalenberg K., Duarte J.M., Dutta S. (2019). RCSB Protein Data Bank: Biological macromolecular structures enabling research and education. Nucleic Acids Res..

[24] Dassault Systèmes BIOVIA (2015). Discovery Studio Modeling Environment, Release 4.5.

[25] Kaya S., von Szentpály L., Serdaroğlu G., Guo L. (2023). Chemical Reactivity: Theories and Principles.

[26] Gürer E.S., Kaya S., Berisha A., Morales-Bayuelo A. (2025). On the effect of conceptual density functional theoretical descriptors in the inhibition of acetylcholinesterase enzyme: Gallic acid derivatives. J. Mol. Struct..

[27] Kaya S., Kaya C., Obot I.B., Islam N. (2016). A novel method for the calculation of bond stretching force constants of diatomic molecules. Spectrochim. Acta Part A Mol. Biomol. Spectrosc..

[28] Kaya S., Robles-Navarro A., Mejía E., Gómez T., Cardenas C. (2022). On the prediction of lattice energy with the Fukui potential: Supports on hardness maximization in inorganic solids. J. Phys. Chem. A.

[29] Pearson R.G. (1988). Absolute electronegativity and hardness: Applications to inorganic chemistry. Inorg. Chem..

[30] Pearson R.G. (1985). Absolute electronegativity and absolute hardness of Lewis acids and bases. J. Am. Chem. Soc..

[31] Parr R.G., Szentpály L.V., Liu S. (1999). Electrophilicity index. J. Am. Chem. Soc..

[32] Koopmans T. (1934). Über die Zuordnung von Wellenfunktionen und Eigenwerten zu den einzelnen Elektronen eines Atoms. Physica.

[33] De Proft F., Van Alsenoy C., Peeters A., Langenaeker W., Geerlings P. (2002). Atomic charges, dipole moments, and Fukui functions using the Hirshfeld partitioning of electron density. J. Comput. Chem..

[34] Morales-Bayuelo A., Vivas-Reyes R., Kaya S. (2024). Analyzing ligands against malaria through docking, molecular similarity and reactivity indices. Front. Chem..

[35] Frisch M.J., Trucks G.W., Schlegel H.B., Scuseria G.E., Robb M.A., Cheeseman J.R., Scalmani G., Barone V., Petersson G.A., Nakatsuji H. (2016). Gaussian 16, Rev. B.01.

[36] Giustarini D., Dalle-Donne I., Milzani A., Fanti P., Rossi R. (2013). Analysis of GSH and GSSG after derivatization with N-ethylmaleimide. Redox Biol..

[37] Ghelfi A., Gaziola S.A., Cia M.C., Chabregas S.M., Falco M.C., Kuser-Falcão P.R., Azevedo R.A. (2011). Cloning, expression, modelling and docking of glutathione transferase from Saccharum officinarum. J. Biotechnol..

[38] Aebi H. (1974). Catalase. Methods of Enzymatic Analysis.

[39] Decker T., Lohmann-Matthes M.L. (1988). A quick and simple method for the quantitation of lactate dehydrogenase release. J. Immunol. Methods.

[40] Smyth E.C., Nilsson M., Grabsch H.I., van Grieken N.C., Lordick F. (2020). Gastric cancer. Lancet.

[41] Choi S. (2012). Encyclopedia of Signaling Molecules.

[42] Sun D., Qian H., Li J., Xing P. (2024). Targeting MDM2 in malignancies to overcome immunotherapy resistance. Signal Transduct. Target. Ther..

[43] Dang C.V. (2012). MYC on the path to cancer. Cell.

[44] Gong J., Chehrazi-Raffle A., Reddi S., Salgia R. (2018). Development of PD-1 and PD-L1 inhibitors as cancer immunotherapy. J. Hematol. Oncol..

[45] Tahiroğlu V., Gören K., Bağlan M. (2025). In silico drug evaluation by docking, ADME and DFT analysis of imidazo [1,2-a]pyridine derivative. Comput. Biol. Chem..

[46] Tahiroğlu V., Gören K., Kotan G., Yüksek H. (2025). In silico drug evaluation and synthesis, characterization of novel Mannich bases. J. Mol. Struct..

[47] Çimen E., Gören K., Tahiroğlu V., Yıldıko Ü. (2025). DFT, ADME and docking study of pyrimidine-thiones compound. J. Mol. Struct..

[48] Mermer A., Alyar S. (2022). Synthesis, DFT and docking of thiosemicarbazide derivatives and Cu(II) complexes. J. Mol. Struct..

[49] Gören K., Yıldıko Ü. (2024). ADME and docking of spiroindoline derivative against aldose reductase. Chem. Biol. Interact..

[50] Gören K. (2024). Molecular docking and theoretical analysis of phenylthiazolidinone derivative. Front. Chem..

[51] Kaya S., Kaya C. (2015). A new method for calculation of molecular hardness: A theoretical study. J. Mol. Graph. Model..

[52] Kaya S., Kaya C. (2015). A new equation for calculation of chemical hardness of groups and molecules. Mol. Phys..

[53] Pearson R.G. (1963). Hard and soft acids and bases. J. Am. Chem. Soc..

[54] Pearson R.G. (1993). The principle of maximum hardness. Acc. Chem. Res..

[55] Chamorro E., Chattaraj P.K., Fuentealba P. (2003). Variation of the electrophilicity index along the reaction path. J. Phys. Chem. A.

[56] Cinar G., Agbektaş T., Huseynzada A., Aliyeva G., Aghayev M., Hasanov U., Kaya S., Chitov S., Nour H., Taş A. (2023). Effect of azomethine macroheterocycles on oxidative stress and DNA repair gene profiles. Bioorg. Chem..

[57] Purushotham M., Paul B., Govindachar D.M., Singh A.K., Periyasamy G., Peter S.C. (2022). Halogen effects and chiral attributes in N-aryl glycine peptoid foldamers. J. Mol. Struct..

[58] Poša M. (2024). The Gibbs-Helmholtz equation and EEC phenomenon in surfactant micellization. J. Mol. Liq..

[59] Le Borgne M., Falson P., Boumendjel A. (2023). Drug candidates targeting multidrug resistance in cancer. Eur. J. Med. Chem..

[60] Gu L., Chen M., Guo D., Zhu H., Zhang W., Pan J., Zhong X., Li X., Qian H., Wang X. (2017). PD-L1 and gastric cancer prognosis: A systematic review and meta-analysis. PLoS ONE.

[61] Zhang M., Dong Y., Liu H., Wang Y., Zhao S., Xuan Q., Wang Y., Zhang Q. (2016). PD-L1 expression and gastric cancer prognosis: Meta-analysis. Oncotarget.

[62] Xu F., Feng G., Zhao H., Liu F., Xu L., Wang Q., An G. (2015). Clinicopathologic significance of B7-H1 in gastric cancer. Medicine.

[63] Bellini M.F., Cadamuro A.C.T., Succi M., Proença M.A., Silva A.E. (2012). Alterations of the TP53 gene in gastric and esophageal carcinogenesis. Biomed. Res. Int..

[64] Carson D.A., Lois A. (1995). Cancer progression and p53. Lancet.

[65] Calcagno D.Q., Leal M.F., Assumpção P.P., Smith M.A.C., Burbano R.R. (2008). MYC and gastric adenocarcinoma carcinogenesis. World J. Gastroenterol..

[66] Zhang L., Hou Y., Ashktorab H., Gao L., Xu Y., Wu K., Zhai J., Zhang L. (2010). Impact of C-MYC expression on gastric cancer cells. Mol. Cell. Biochem..

